# Isolation and Characterization of a High-Affinity Anti-TSPAN-1 scFv Antibody from a Phage-Display Library for Planarian Neoblast Targeting

**DOI:** 10.3390/biology15211928

**Published:** 2026-10-24

**Authors:** Yanan Guo, Wanru Li, Yiyi Zhao, Shoutao Zhang, Weiyu Zhao

**Affiliations:** 1 School of Life Sciences, Shanghai University, No. 99 Shangda Road, Shanghai 200444, China; zyy11@shu.edu.cn; 2 School of Life Sciences, Zhengzhou University, Zhengzhou 450001, China; zzhouu2026@163.com (W.L.); zhangst@zzu.edu.cn (S.Z.); 3 Logistics Engineering College, Shanghai Maritime University, Shanghai 201306, China; 4 SILC Business School, Shanghai University, Shanghai 201899, China

**Keywords:** single-chain variable fragment, phage display, TSPAN-1, neoblasts, planarian

## Abstract

Background: Planarian neoblasts are pluripotent adult stem cells responsible for whole-body regeneration, yet antibody-based tools for their specific identification and isolation remain limited. This study aimed to generate a high-affinity single-chain variable fragment (scFv) targeting TSPAN-1, a neoblast surface marker, using phage display technology. Methods: A phage-displayed scFv library (3.86 × 10^7^ CFU) was constructed from mice immunized with the recombinant large extracellular loop (LEL) of TSPAN-1 fused to maltose-binding protein (MBP). Four rounds of bio-panning were performed against immobilized His-LEL. Positive clones were screened by ELISA, colony PCR, and DNA sequencing. Among the characterized clones, scFv-16 showed the highest apparent affinity. This clone was subcloned for prokaryotic expression, and inclusion body proteins were refolded and purified. Results: Friguet analysis gave an apparent affinity constant (Kaff) of 4.20 × 10^8^ M^−1^ for scFv-16 binding to His-TSPAN-1. This corresponds to a KD of 2.38 nM. Western blotting and immunofluorescence staining supported the specificity of scFv-16 for TSPAN-1. Conclusions: A high-affinity anti-TSPAN-1 scFv was successfully generated, providing a candidate molecular tool for neoblast identification and potential isolation in planarian regeneration studies.

## Simple Summary

Planarian neoblasts are pluripotent adult stem cells that sustain whole-body regeneration, yet reliable tools for their specific labeling and isolation remain limited. Here, we employed a phage-display library to screen and identify a high-affinity single-chain variable fragment (scFv) antibody targeting planarian TSPAN-1—a cell-surface marker of planarian neoblasts. The anti-TSPAN-1 scFv was characterized for binding affinity and specificity, supporting its use as a candidate molecular probe for neoblast identification and potential isolation. As a practical addition to the planarian toolkit, this scFv is expected to facilitate future studies of neoblast biology and regeneration.

## 1. Introduction

Pluripotent stem cells (PSCs) are ideal in vitro models for deciphering embryonic development, cell lineage commitment, and organogenesis, and serve as crucial tools for elucidating the mechanisms of ontogeny and aging [1,2]. Unraveling their biological functions and molecular regulatory networks is continuously expanding the therapeutic frontiers for treating intractable diseases and promoting in situ tissue repair [3,4]. However, naturally occurring adult pluripotent stem cells have yet to be identified in traditional model organisms such as *Caenorhabditis elegans*, *Drosophila*, *zebrafish*, and mice [5,6]. In contrast, planarians harbor an abundant population of adult stem cells, collectively termed neoblasts. Among these, clonogenic neoblasts, a subpopulation of pluripotent stem cells, endow planarians with remarkable regenerative capabilities and a virtually unlimited capacity to maintain tissue homeostasis [7,8,9]. Consequently, planarians provide a unique and indispensable model system for addressing a fundamental scientific question: how stem cell pluripotency is sustained long-term in adult animals [10]. Although significant progress has been achieved in the short-term in vitro culture of planarian neoblasts, the lack of long-term in vitro maintenance systems severely hinders the investigation of their pluripotency regulatory mechanisms and the development of transgenic tools [11,12].

Molecular markers for planarian neoblasts are broadly classified into intracellular and cell-surface markers [13,14]. The canonical intracellular marker is Smedwi-1 (also known as PIWI-1), a member of the PIWI/Argonaute protein family [15,16]. Conversely, TSPAN-1 (also referred to as P503s, C4.8, or NET-1) serves as a prominent cell-surface marker [17]. As a member of the tetraspanin superfamily, TSPAN-1 features a characteristic topology comprising four transmembrane domains, a short intracellular loop, and two unequal extracellular loops [18,19]. Notably, the large extracellular loop (LEL), situated between the third and fourth transmembrane domains, constitutes the primary functional region of the protein. The LEL can be further subdivided into five conserved domains (A, B, C, D, and E), which play crucial roles in protein–protein interactions, cell adhesion, and signal transduction [20,21].

Phage display technology establishes a direct physical linkage between the antibody phenotype and its encoding genotype by displaying the single-chain variable fragment (scFv) as a fusion protein with a phage coat protein (pIII or pVIII) [22,23]. This paradigm facilitates the rapid enrichment of high-affinity and highly specific scFvs from vast repertoires via bio-panning, thereby circumventing the inherent limitations of traditional hybridoma technology, namely restricted repertoire diversity and lengthy development cycles [24,25,26]. Furthermore, the platform readily supports in vitro affinity maturation; through directed evolution strategies such as error-prone PCR and DNA shuffling, binding affinities can be enhanced to the picomolar (pM) range [27,28,29]. Collectively, these attributes have established phage display as a cornerstone technology in recombinant antibody engineering, molecular diagnostics, therapeutic antibody discovery, and tumor immunotherapy [25,30].

In this study, we targeted TSPAN-1, a defining surface marker of planarian neoblasts. To generate a specific immunogen, the gene encoding the large extracellular loop (LEL) of TSPAN-1 was cloned and expressed in *Escherichia coli*, followed by affinity purification. This recombinant protein was used to immunize mice, and a murine immune phage-displayed scFv library was constructed. Four rounds of bio-panning against immobilized His-TSPAN-1 were then performed. Clones binding TSPAN-1 were identified by ELISA, colony PCR, and DNA sequencing. Among the characterized clones, scFv-16 showed the highest apparent affinity; it was expressed in *E. coli*, refolded from inclusion bodies, and purified. This high-affinity scFv is a candidate molecular tool for neoblast identification. Its application to live-cell sorting and in vitro neoblast culture requires further experimental validation and remains a direction for future work.

## 2. Materials and Methods

### 2.1. Materials

Tetramethylbenzidine (TMB, #T4444), Freund’s complete and incomplete adjuvant (FCA, #F5881 and FIA, #F5506) were purchased from Sigma-Aldrich (St. Louis, MO, USA). Molecular biology enzymes, including restriction enzymes and T4 DNA ligase (#M0202V), were obtained from New England Biolabs (Beijing, China). The BCA protein assay kit (#CW0014S), HRP-conjugated anti-His-tag mouse monoclonal antibody (#CW0285M), and HRP-labeled goat anti-rabbit (#CW0103S) and goat anti-mouse secondary antibodies (#CW0102S) were supplied by CoWin Biosciences (Beijing, China). Nucleic acid manipulation kits (DNA gel extraction, #DP209 and plasmid mini-prep, #DP103) were acquired from Tiangen Biotech (Beijing, China). PCR master mixes (2× Taq, #TSE004 and 2× high-fidelity, #DLP202) were from Tsingke Biotechnology (Beijing, China). Skim milk powder was obtained from Solarbio (Beijing, China, #SW3013).

### 2.2. Recombinant Protein Expression and Purification

The *Schmidtea mediterranea* cDNA was generously provided by Dr. Shoutao Zhang’s laboratory (Zhengzhou University). To prepare the immunogen, we amplified the gene fragment encoding the large extracellular loop (LEL) of *TSPAN-1* (GenBank accession No. MG551539.1) using specific primers (TSPAN-1 F/R) and cloned it into the *Nde*I/*EcoR*V sites of the pMAL-C5X vector, yielding the pMAL-C5X-LEL construct. After Sanger sequencing verification, the recombinant plasmid was transformed into *Escherichia coli* BL21(DE3) to express the MBP-LEL fusion protein. The recombinant protein was affinity-purified according to our previously described protocol [31], and its purity was analyzed by SDS-PAGE. For panning and binding assays, the LEL coding sequence of TSPAN-1 was amplified (primers pc-TSPAN-1 F/R) and subcloned into the *BamH*I/*EcoR*V sites of the pcDNA3.1(+) vector to yield pcDNA3.1-LEL.Upon sequence confirmation, the plasmid was transfected into HEK 293T cells (RRID, CVCL_0063) (provided by Dr. Shoutao Zhang at Zhengzhou University) to express the His-tagged LEL (His-LEL) protein. His-LEL protein was subsequently affinity-purified using the established methods and evaluated by SDS-PAGE. Primers are listed in Supplementary Table S1.

### 2.3. Animal Immunization

The Ethics Committee of Zhengzhou University authorized all animal studies (approval no. ZZUIRB2025-103, Approval Date: 14 March 2025), which were performed under specific pathogen-free conditions in compliance with institutional and national guidelines. For the primary immunization, three 8-week-old female specific pathogen-free (SPF) BALB/c mice (Henan Experimental Animal Center, Zhengzhou, China) were immunized with an emulsion containing 50 μg of MBP-LEL (100 μL at 0.5 mg/mL, Section 2.2) and 100 μL of Freund’s complete adjuvant (FCA). Pre-immune sera were collected one week prior to immunization. Booster immunizations were administered biweekly with the identical antigen dose emulsified in Freund’s incomplete adjuvant (FIA). One week after the third immunization, blood samples were collected from the tail vein, and serum anti-TSPAN-1 antibody titers were determined via indirect ELISA (Section 2.4). The mouse exhibiting the highest titer received a final booster injection of 10 μg of MBP-LEL in PBS without adjuvant prior to splenocyte isolation.

### 2.4. Determination of Antiserum Titer

Antiserum titers were determined via indirect ELISA. Briefly, 96-well microplates were coated overnight at 4 °C with 100 μL/well of MBP-LEL (0.5 mg/mL). The plates were then blocked with 200 μL/well of 5% (*w*/*v*) skim milk in PBS (MPBS) for 1 h at 37 °C. Subsequently, 100 μL of serially diluted antiserum samples in PBS (starting at a 1:5000 dilution) was added per well and incubated for 1 h at 37 °C. After washing, 100 μL of HRP-conjugated goat anti-mouse IgG (1:5000 dilution) was added and incubated for 1 h at 37 °C. Unless otherwise specified, the plates were washed three times with PBST (PBS containing 0.05% Tween-20) between each incubation step. Color development was performed using 100 μL/well of TMB substrate in the dark for 15 min at 37 °C. The reaction was terminated with 100 μL/well of stop solution (1 M H_2_SO_4_), and the absorbance was recorded at 450 nm using a microplate reader.

### 2.5. Construction of the scFv Library

The scFv library was constructed following established protocols with minor modifications [32]. Total RNA was extracted from murine splenic tissues using TRIzol reagent (Invitrogen, Carlsbad, CA, USA), and its quality and concentration were assessed via a NanoDrop spectrophotometer (Implen, Munich, Germany). First-strand cDNA was synthesized using the SuperScript^®^ III First-Strand Synthesis System (Invitrogen, Carlsbad, CA, USA) according to the manufacturer’s instructions. Using the cDNA as a template, the heavy chain variable region (VH) was amplified with primers VH-F and VH-R. In contrast, the light chain variable region (VL) was amplified with forward primer VL-F and a reverse primer mix (an equimolar mixture of VL-R1, VL-R2, VL-R3, and VL-R4). Thermocycling conditions for both amplifications included an initial denaturation at 98 °C for 2 min, followed by 32 cycles of 98 °C for 10 s, 55 °C for 30 s, and 68 °C for 15 s, with a final extension at 68 °C for 5 min. The PCR products were verified by agarose gel electrophoresis and subsequently purified. Equimolar amounts of the purified VH, VL, and linker fragments were assembled into full-length scFv genes via splice overlap extension PCR (SOE-PCR). The initial primer-free assembly comprised a denaturation step at 98 °C for 2 min, followed by 10 cycles of 98 °C for 10 s, 55 °C for 30 s, and 68 °C for 30 s, and a final extension at 68 °C for 5 min. Subsequently, the flanking primers VH-SfiI-F and VL-NotI-Rmix (an equimolar mixture of VL-NotI-R1 to R4) were added, and amplification was continued for an additional 32 cycles under identical conditions. The amplified scFv fragments and the pCANTAB5E phagemid vector were double-digested with SfiI and NotI, gel-purified, and ligated using T4 DNA ligase. The recombinant phagemids were then electroporated into *Escherichia coli* TG1 electrocompetent cells. To determine the library size, serial 10-fold dilutions of the recovered electroporated cells were plated on selective agar, and the total number of colony-forming units (CFUs) was calculated, yielding a library size of 3.86 × 10^7^ CFU. To preliminarily assess library diversity, nine individual colonies were randomly selected for colony PCR using primers p5F and p5R, followed by Sanger sequencing. The nine sequences differed from one another and were not derived from a single parental clone. A comprehensive assessment of library diversity will require deep sequencing. The remaining transformants were harvested by flooding the plates with 2×YT-A medium (16 g/L tryptone, 10 g/L yeast extract, and 5 g/L NaCl, supplemented with 100 μg/mL ampicillin) and gently scraping the cells with a sterile spreader. The collected bacterial suspension was mixed with glycerol to a final concentration of 15% (*v*/*v*) and stored at −80 °C as the primary scFv library.

### 2.6. Bio-Panning and Identification of Enriched scFvs

Phage library bio-panning was performed according to established protocols [33]. Briefly, 96-well microplates were coated overnight at 4 °C with His-LEL (100 μL/well, 50 μg/mL). Following coating, wells were blocked with 5% MPBS (200 μL/well) for 1 h at 37 °C. After three washes with PBST (PBS containing 0.1% Tween-20), the phage library (100 μL/well) was added and incubated for 2 h at room temperature (RT). Unbound phages were removed by 10 stringent washes with PBST. Bound phages were then eluted with 0.2 M glycine-HCl (pH 2.2, 100 μL/well) for 10 min at RT. The eluate was immediately neutralized with an equal volume of 1 M Tris-HCl (pH 7.4) to yield the first-round output. The neutralized eluate was used to infect log-phase *E. coli* TG1. The infected culture was grown to OD600 ≈ 0.5 in 2×YT-A with 2% glucose and rescued with helper phage M13-K07 (MOI ≈ 20:1) for 30 min at 37 °C, then cultured overnight in 2×YT-AK (2×YT-A with 50 μg/mL kanamycin) at 30 °C. Phages in the cleared supernatant were PEG/NaCl-precipitated, resuspended in PBS, and used as the input for the next round of panning; input and output titers were determined each round by plating serial dilutions on TG1. Enrichment was calculated as the ratio of the output phage titer in round 4 to that in round 1. To progressively increase the selection stringency, the His-LEL coating concentration was decreased to 25 μg/mL in the second round, and to 10 μg/mL and 5 μg/mL in the third and fourth rounds, respectively. Additionally, the Tween-20 concentration in the wash buffer was increased to 0.5% (*v*/*v*) during the latter two rounds. After the fourth round, an aliquot of the eluate was used to determine phage titer, while the remainder was plated on TYE-AG agar and incubated overnight at 37 °C. Colonies randomly picked from the fourth-round output plates were grown in 96-deep-well plates (200 μL of 2×YT-A with 0.1% glucose per well) at 37 °C to OD600 ≈ 0.5 and infected with helper phage M13-K07 (MOI ≈ 20:1). After overnight culture in 2×YT-AK at 30 °C, the supernatants containing monoclonal phages were collected for ELISA screening.

Microplate wells were coated overnight at 4 °C with His-LEL (5 μg/mL). Wells incubated with helper phage M13-K07 (no scFv insert) served as the negative control in each group. The plates were blocked with 5% MPBS, and incubated with monoclonal phage supernatants for 2 h at RT, with PBST washes between steps. Bound phages were detected with an HRP-conjugated anti-M13 antibody (0.4 μg/mL, Sino Biological, Beijing, China, Cat#11973-MM05T-H, RRID: AB_2857928), developed the reaction with TMB, and the reaction was stopped with 1 M H_2_SO_4_. Absorbance at 450 nm was measured. Each clone was measured in triplicate. Positivity was defined as an OD450 value above the mean plus three standard deviations of the M13-K07 control wells. Data were background-subtracted without normalization, and all clones were screened on a single plate. Phagemids from clones exhibiting positive signals in colony PCR, monoclonal ELISA, and double restriction digestion were extracted and sequenced. Sequence homology was evaluated using the NCBI BLAST database (version 2.17.0), and the framework regions (FRs) and complementarity-determining regions (CDRs) of the VH and VL domains were delineated using the IgBLAST tool (version 1.20.0). The physicochemical properties of the deduced scFv proteins were predicted via the ExPASy ProtParam server (https://web.expasy.org/protparam/; accessed on 23 October 2026). Finally, glycerol stocks of the bacterial cultures harboring the verified sequences were prepared at a final concentration of 25% (*v*/*v*) and stored at −80 °C.

### 2.7. Expression and Binding Analysis of Murine scFvs

The DNA sequences encoding the candidate murine scFvs were cloned into the pET-15b vector via the *BamH*I and *Nde*I restriction sites. The resulting recombinant plasmids were then transformed into *Escherichia coli* BL21(DE3) competent cells. A single colony was inoculated into 2×YT-A medium (16 g/L tryptone, 10 g/L yeast extract, and 5 g/L NaCl, supplemented with 100 μg/mL ampicillin) and cultured overnight at 37 °C with shaking. The overnight culture was subcultured into fresh 2×YT-A medium and incubated at 37 °C until the optical density at 600 nm (OD600) reached 0.6–0.8. Recombinant protein expression was induced by the addition of isopropyl-β-D-thiogalactopyranoside (IPTG) to a final concentration of 1 mM, followed by overnight incubation at 16 °C and 180 rpm. The scFvs were expressed as inclusion bodies. They were refolded in vitro and purified as previously described [34], then buffer-exchanged into PBS. For binding affinity analysis, 96-well microplates were coated overnight at 4 °C with His-LEL at two concentrations (2 and 1 μg/mL). After three washes with PBST, the wells were blocked with 5% MPBS for 1 h at 37 °C. To determine the apparent affinity constant (Kaff) of the scFvs binding to His-LEL, 100 μL of purified scFv protein was added to each well in 2-fold serial dilutions (starting at 2 μg/mL). Following incubation and washing, bound scFvs were detected using an HRP-conjugated anti-His-tag mouse monoclonal antibody. The absorbance data were fitted to a four-parameter logistic curve in GraphPad Prism 8.0. The scFv concentration at half-maximal absorbance was determined for each antigen concentration, and the apparent Kaff was calculated according to the method of Friguet et al. [35].

### 2.8. Binding Analysis of scFv Fragments to the TSPAN-1 Antigen

The binding of the scFv fragments was assessed at three levels: recombinant TSPAN-1 antigens by Western blot, endogenous TSPAN-1 in planarian lysates by Western blot, and neoblasts in planarian tissues by immunofluorescence. For the recombinant antigens, Purified MBP-LEL, His-LEL, and MBP (negative control) were resolved by SDS-PAGE and transferred to membranes. The blots were probed with the purified scFv proteins, followed by detection using an HRP-conjugated mouse anti-His-tag monoclonal secondary antibody. Specific binding was assessed by the detection of distinct immunoreactive bands at approximately 51 kDa (MBP-LEL) and 12 kDa (His-LEL), with no cross-reactivity observed against the MBP control. To examine endogenous TSPAN-1, total protein was extracted from *Schmidtea mediterranea*, and Western blotting was performed with purified scFv-16 as the primary antibody (diluted 1:1000). A single band appeared at approximately 28 kDa, consistent with the theoretical molecular weight of planarian TSPAN-1. For immunofluorescence, Planarians were starved, rinsed in DEPC-treated water, and treated with 2% HCl at room temperature (RT) to induce muscle relaxation prior to fixation. Specimens were fixed in 4% paraformaldehyde (PFA), dehydrated in 100% methanol at −20 °C for 1 h, and permeabilized with 1% SDS at RT for 30 min. Following rehydration through a graded methanol series (75%, 50%, and 25% in PBST) and three PBST washes, the samples were blocked with 10% skim milk at RT for 2 h. The samples were incubated overnight at RT under gentle agitation with the scFv proteins together with rabbit anti-H3P (phospho-histone H3 Ser10, Proteintech, Rosemont, IL, USA, Cat# 82828-18-RR, RRID: AB_3743439) as an independent marker of dividing neoblasts [36,37]. After a 2.5 h wash in PBST, the samples were incubated with a FITC-conjugated anti-His-tag secondary antibody and an Alexa Fluor 568-conjugated anti-rabbit secondary antibody overnight at RT in the dark. A final 2.5 h wash in PBST followed, and the specimens were visualized and imaged with an inverted fluorescence microscope (Olympus, Tokyo, Japan).

## 3. Results

### 3.1. Preparation of Recombinant Proteins

TSPAN-1 is a defining surface marker for planarian neoblasts [17]. Its large extracellular loop (LEL) is exposed to the extracellular milieu and enriched in hydrophilic residues, conferring high antigenic accessibility and making it an ideal target for antibody development.

**Figure 1:**
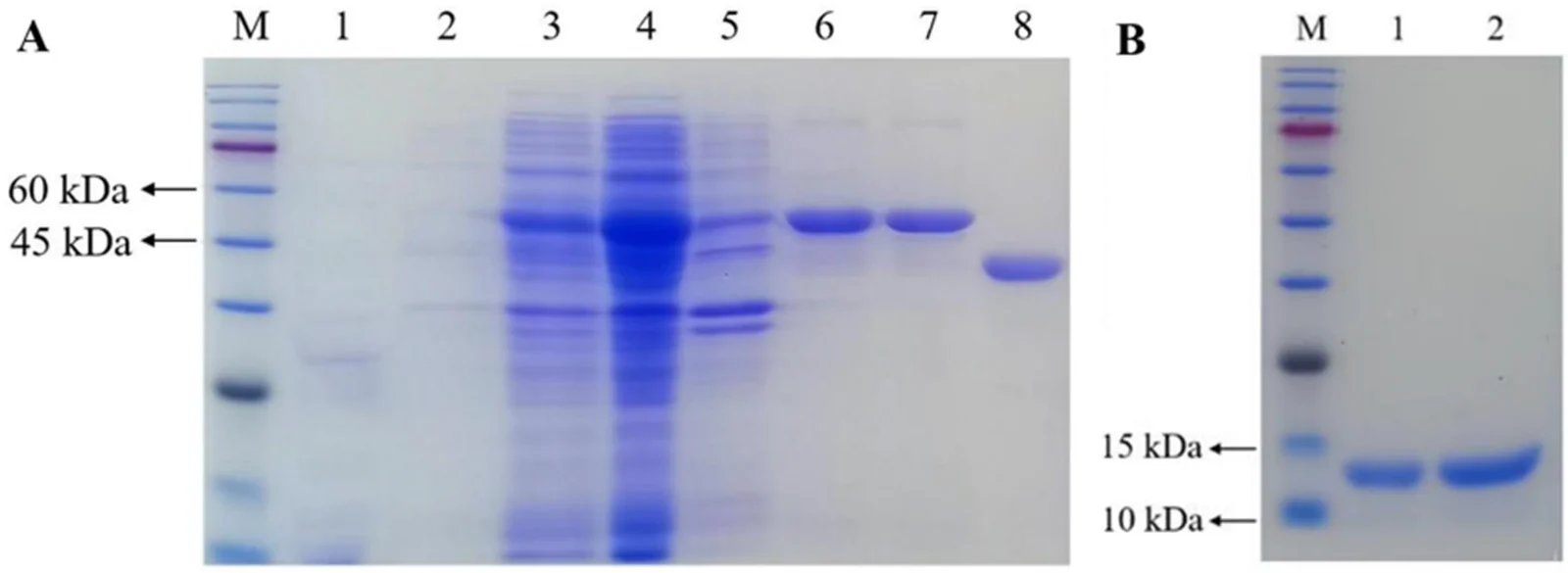
SDS-PAGE analysis of purified fusion proteins. (**A**) Purification of MBP-LEL. Lane M, protein marker (10–180 kDa); lane 1, whole-cell lysate of BL21(DE3) (uninduced); lane 2, whole-cell lysate of recombinant BL21(DE3) before induction; lane 3, whole-cell lysate of recombinant BL21(DE3) after IPTG induction; lane 4, clarified lysate (input); lane 5, flow-through fraction; lanes 6–7, elution fractions; lane 8, purified MBP tag protein. (**B**) Purification of His-LEL. Lane M, protein marker (10–180 kDa); lanes 1–2, purified His-LEL protein.

In this study, the TSPAN-1 LEL was utilized as the target antigen to isolate anti-TSPAN-1 single-chain variable fragments (scFvs) via phage display. To optimize cost-effectiveness and experimental efficiency, two distinct recombinant antigen formats were strategically generated for animal immunization and phage bio-panning, respectively. A 321 bp DNA fragment encoding the TSPAN-1 LEL was amplified from *Schmidtea mediterranea* cDNA (Figure S1). The amplicon was double-digested with *Nde*I and *EcoR*V and cloned into the pMAL-C5X vector to construct the prokaryotic expression plasmid pMAL-C5X-LEL. Following sequence verification, the recombinant plasmid was transformed into *Escherichia coli* BL21(DE3) for the IPTG-induced expression of the MBP-LEL fusion protein. The target protein was purified via affinity chromatography, and its expression and purity were confirmed by SDS-PAGE (Figure 1A).

Simultaneously, to prepare the bio-panning target, the TSPAN-1 LEL fragment was digested with *BamH*I and *EcoR*V and subcloned into the pcDNA3.1(+) vector to generate the eukaryotic expression construct pcDNA3.1-LEL. Upon sequence confirmation, the plasmid was transfected into HEK293T cells for transient expression. The resulting His-tagged LEL (His-LEL) protein was affinity-purified, and its yield and purity was assessed by SDS-PAGE (Figure 1B).

### 3.2. Animal Immunization and Evaluation of Antiserum Titers

**Figure 2:**
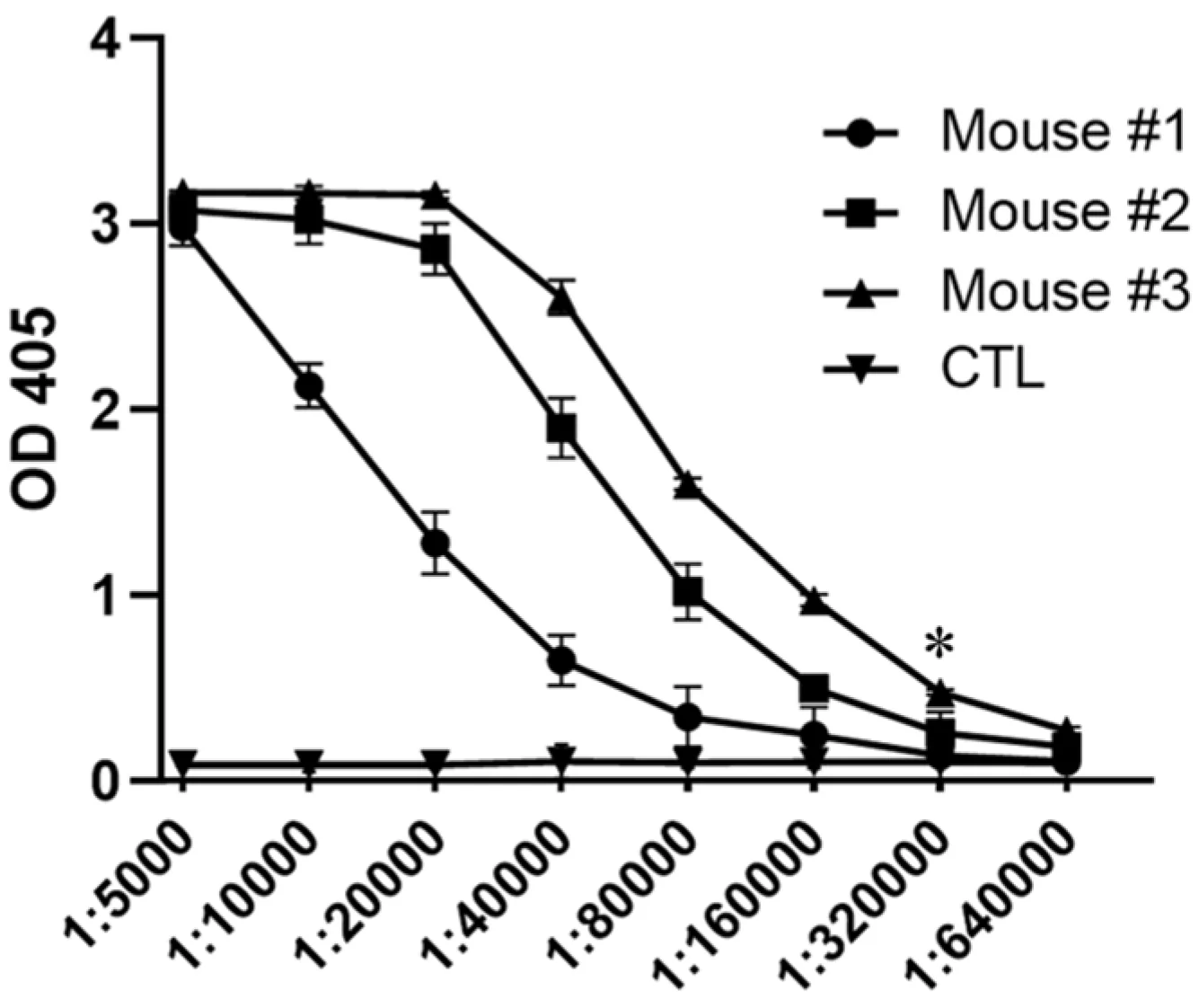
Determination of the binding ability of mouse antisera. Following the final booster injection, the serum titers of the immunized mice were assessed by indirect ELISA against MBP-LEL. The triplicate OD450 values of mouse #3 at the 1:320,000 dilution were compared with those of the negative control using an unpaired two-tailed *t*-test (* *p* < 0.05).

Three female BALB/c mice received a three-dose immunization regimen with purified recombinant MBP-LEL protein. Following the final booster injection, blood samples were collected from the tail veins, and serum anti-TSPAN-1 antibody titers were determined by indirect ELISA. All immunized mice developed robust polyclonal antibody responses against TSPAN-1, with mouse #3 achieving the highest titer of 1:320,000 (Figure 2). Because each mouse is an independent biological unit, mouse-to-mouse differences could not be tested statistically. We therefore compared the triplicate OD450 values of mouse #3 at the 1:320,000 dilution with those of the negative control using an unpaired two-tailed *t*-test. The sample signal was significantly higher (*p* < 0.05). Mouse #3 thus retained a detectable titer at this dilution. Consequently, mouse #3 was selected for splenocyte harvesting and the subsequent construction of the phage-displayed scFv library. These results confirm the strong immunogenicity of the recombinant MBP-LEL protein and its efficacy in eliciting high-titer, antigen-specific antibodies in vivo.

### 3.3. Construction of the Phage Display Library

**Figure 3:**
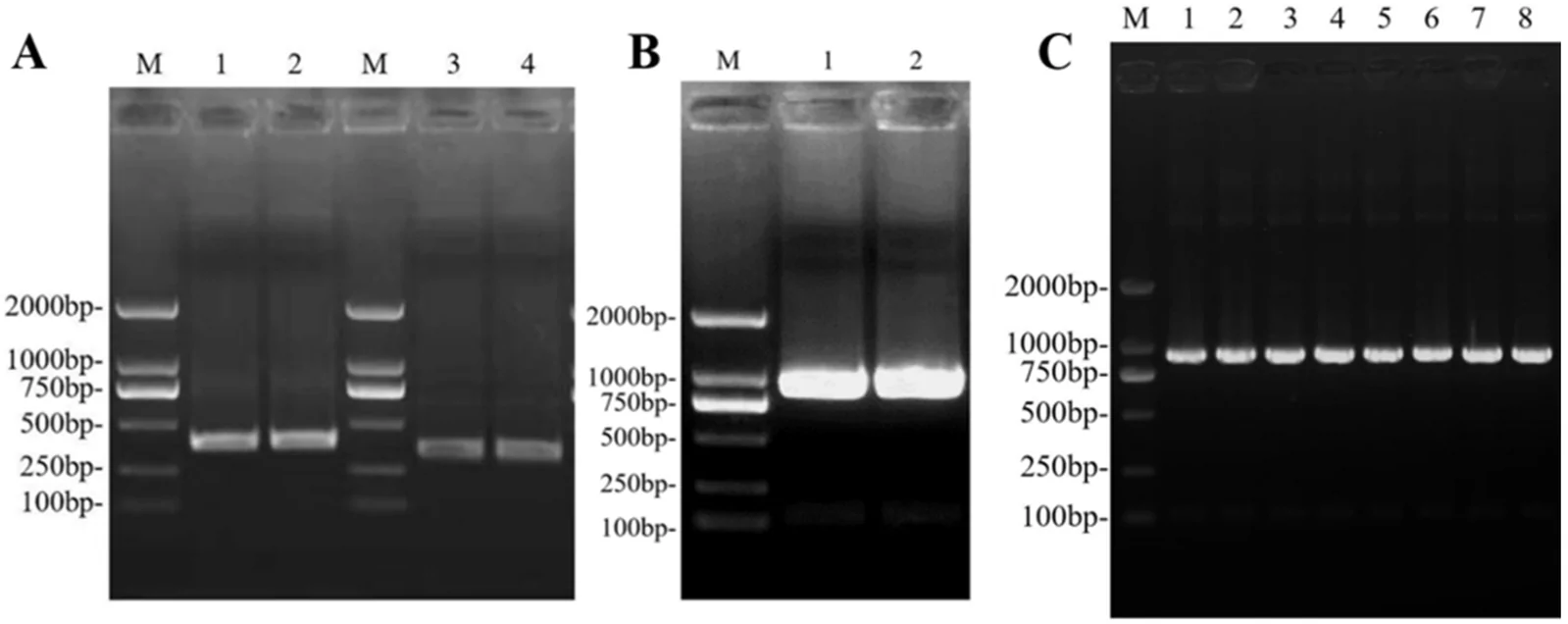
Construction of a phage-display murine scFv library. (**A**) PCR amplification of VH and VL genes. M: DNA marker; lanes 1–2: VH amplicons; lanes 3–4: VL amplicons. (**B**) SOE-PCR assembly and high-fidelity amplification of full-length scFv genes (~750 bp). (**C**) Colony PCR verification of TG1 transformants harboring pCANTAB5E-scFv constructs.

Mouse #3, which exhibited the highest serum antibody titer, was euthanized and its spleen was aseptically harvested for total RNA extraction and reverse transcription into cDNA. Using this cDNA as a template, the VH and VL genes were specifically amplified. Agarose gel electrophoresis revealed single, distinct bands of the expected sizes for both amplicons (Figure 3A), confirming successful amplification of the antibody variable region fragments. After purification, the VH, VL, and flexible linker fragments were assembled via splice overlap extension PCR (SOE-PCR) and subsequently amplified using a high-fidelity DNA polymerase. Agarose gel electrophoresis of the assembled products revealed a single band of the expected size (~750 bp), consistent with the full-length scFv gene (Figure 3B). The purified scFv fragments and the pCANTAB-5E phagemid vector were subjected to double digestion with *Sfi*I and *Not*I, followed by ligation with T4 DNA ligase at 16 °C. The ligation mixture was then electroporated into competent *E. coli* TG1 cells. Colony PCR screening of nine randomly selected clones confirmed that all were positive recombinants (Figure 3C), indicating successful construction of the library. To assess library quality preliminarily, these positive clones were subjected to Sanger sequencing. Sequence analysis confirmed that all inserts were in the correct reading frame and lacked premature stop codons. all nine clones possessed unique sequences, sharing an average nucleotide sequence identity of 79.16%, which indicated that the sampled clones were not derived from a single parental clone. Finally, the library capacity was determined to be 3.86 × 10^7^ CFU by plating the transformed cell suspension on 2×YT-A plates (Figure S2).

### 3.4. Identification of Positive Clones via Binding Assays

**Figure 4:**
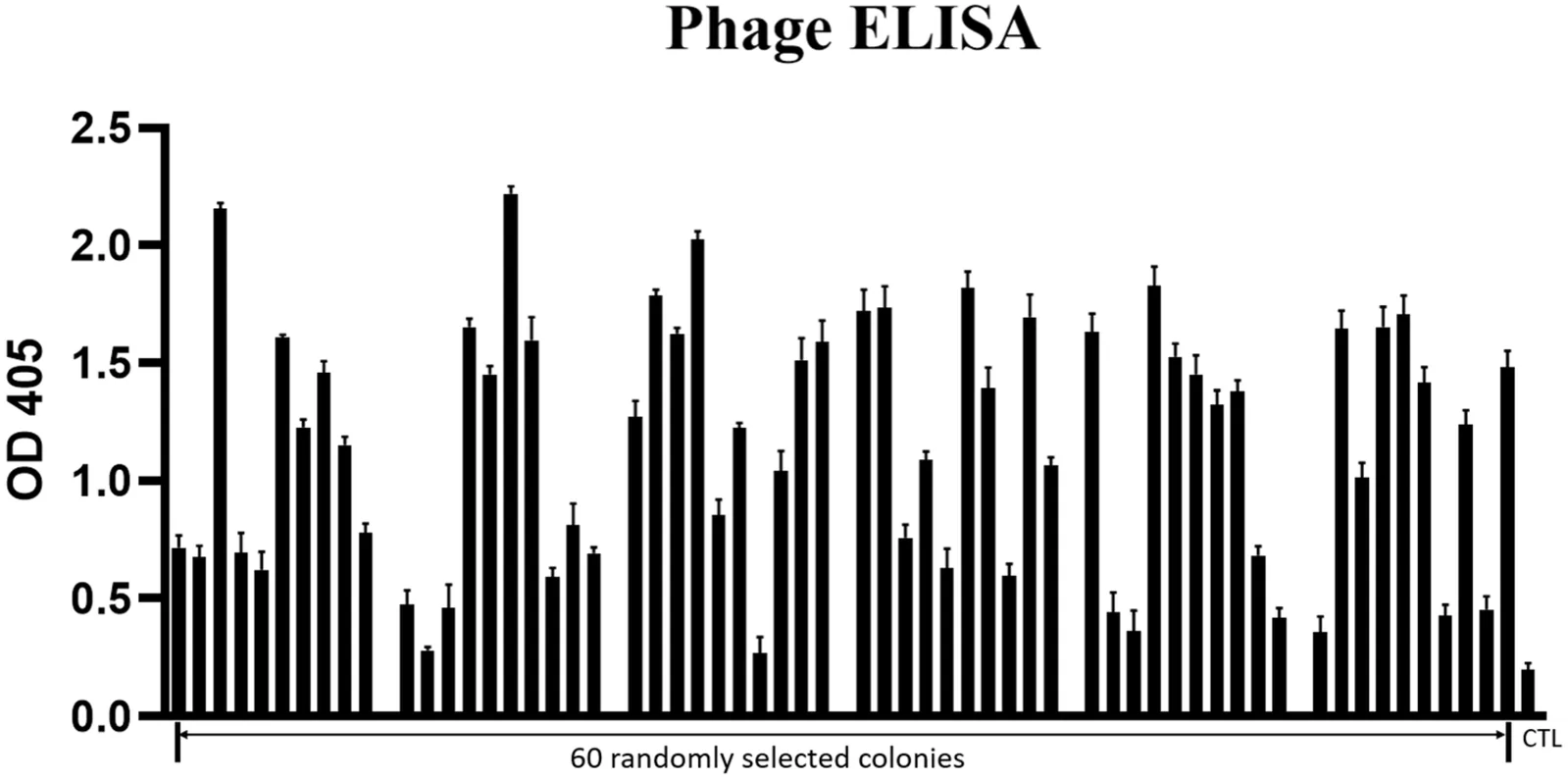
Binding of positive phage-displayed scFv clones from the fourth round of bio-panning to His-LEL. M13-K07 helper phage (no scFv insert) served as the negative control (CTL). Positivity was defined as the M13-K07 mean plus three standard deviations. Each clone was measured in triplicate.

The anti-TSPAN-1 scFv phage library was subjected to four rounds of solid-phase panning against eukaryotically expressed His-LEL. By the fourth round, a 108-fold enrichment was achieved (Table 1), and phage output increased progressively with each round. Enrichment was calculated as the ratio of the output phage titer in round 4 to that in round 1. Because the washing conditions became progressively more stringent across rounds, this increase is consistent with enrichment of target-specific clones, though it does not by itself demonstrate specific enrichment. Following the fourth round, 60 single colonies were randomly selected for colony PCR. Agarose gel electrophoresis revealed a single band of the expected size (~900 bp) for all clones (Figure S3), indicating that the scFv inserts in the pCANTAB-5E-scFv phagemid remained intact without deletions after multiple rounds of panning. The 60 clones were individually cultured and evaluated by phage ELISA. With helper phage M13-K07 (no scFv insert) as the negative control, clones 3, 16, and 24 showed absorbance values above the cutoff (M13-K07 mean + 3 SD) (Figure 4). Recombinant phagemids from these three positive clones were extracted and verified by double digestion with *Not*I and *Sfi*I (Figure S3), which released a ~750 bp insert consistent with the expected scFv size. Sanger sequencing revealed that the full-length sequences of scFv-3, scFv-16, and scFv-24 were 720 bp (encoding 240 amino acids), 732 bp (encoding 244 amino acids), and 726 bp (encoding 242 amino acids), respectively. IgBLAST analysis confirmed that the VH and VL domains of all three scFvs contained the canonical three complementarity-determining regions (CDRs) and four framework regions (FRs), consistent with typical antibody structural features.

### 3.5. Preparation, Purification, and Binding Analysis of scFvs

**Figure 5:**
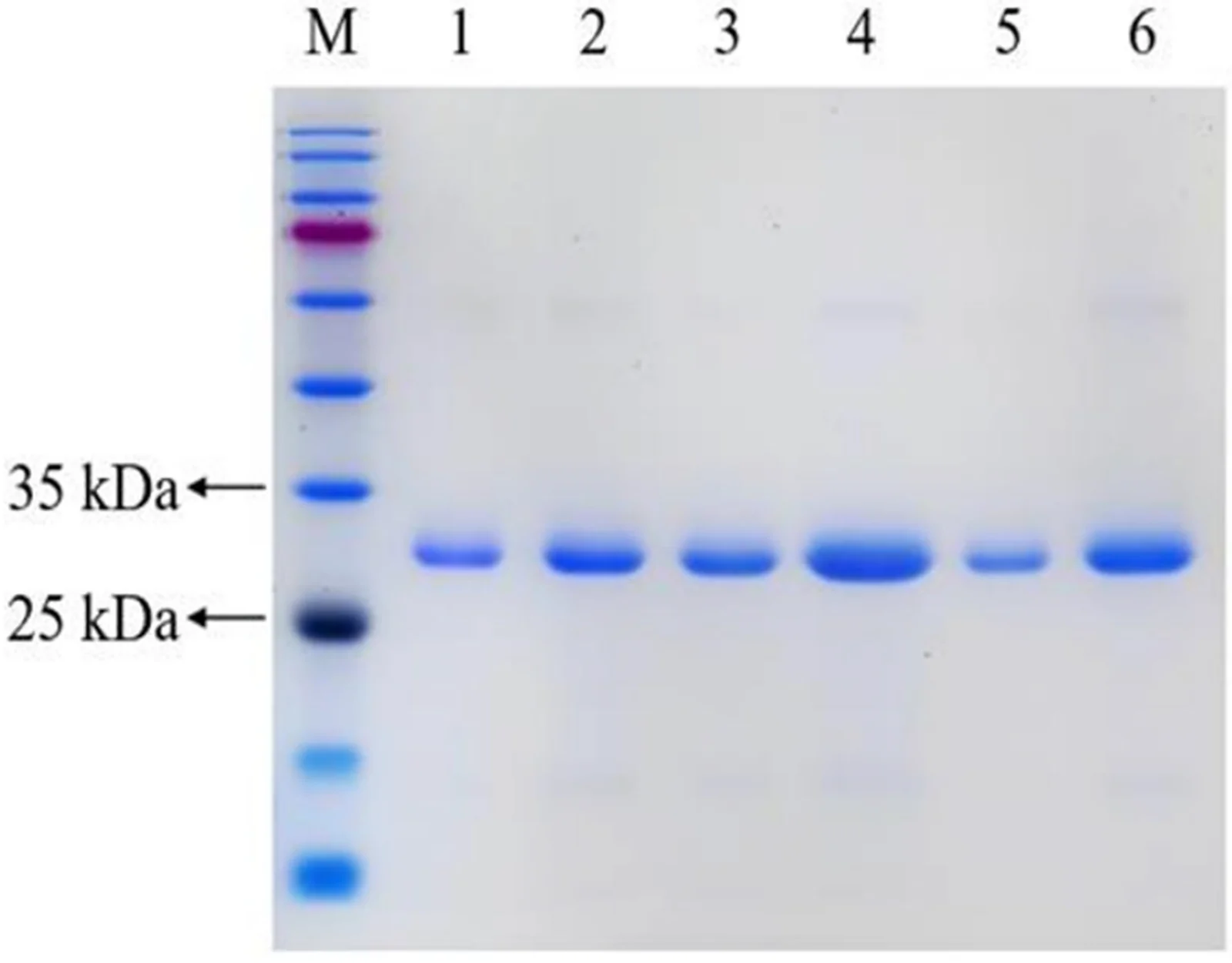
Heterologous expression and purification of recombinant scFv-3, scFv-16, and scFv-24 in *E. coli*. Recombinant *E. coli* strains expressing scFv-3, scFv-16, and scFv-24 were grown in large-scale cultures. Cells were harvested by centrifugation and disrupted by sonication. The inclusion bodies were solubilized in denaturing buffer, and the scFv proteins were purified by Ni^2+^-NTA affinity chromatography, and eluted with imidazole buffer. The eluted proteins were refolded by stepwise dilution and exchanged into PBS. SDS-PAGE revealed a single band of the expected molecular weight for each scFv (lanes 1–2, scFv-3; lanes 3–4, scFv-16; lanes 5–6, scFv-24), indicating that all three scFv proteins were obtained at high purity.

**Figure 6:**
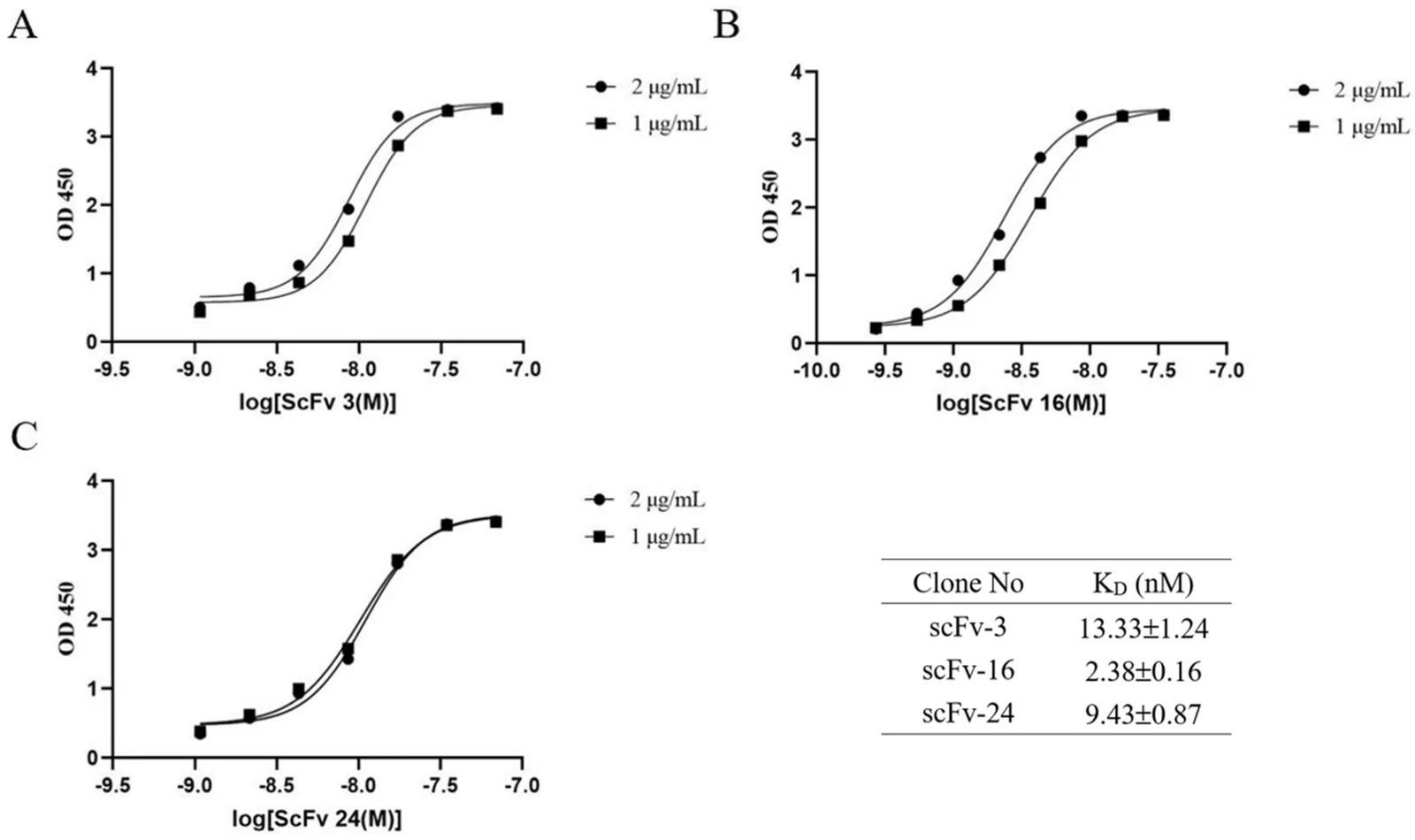
Affinity determination of anti−TSPAN−1 scFvs for His−LEL. The apparent affinities of scFv−3 (**A**), scFv−16 (**B**), and scFv−24 (**C**) were determined by indirect ELISA. Serial dilutions of each scFv were incubated with His-LEL coated at 2 or 1 μg/mL, and the OD450 values were measured. Dose–response curves were fitted using a four-parameter logistic (4PL) model in GraphPad Prism 8.0, and apparent affinity constants (Kaff) were calculated according to Friguet et al. [35]. Summary of the apparent KD values for the three scFvs is shown in the inset table.

The candidate scFv genes from clones 3, 16, and 24 were individually cloned into the pET-15b vector via the *BamH*I and *Nde*I sites to construct prokaryotic expression plasmids (pET-15b-scFv). Following Sanger sequencing verification of the inserts and reading frames, the recombinant plasmids were transformed into competent *E. coli* BL21(DE3) cells for IPTG-induced expression of His-tagged scFv. SDS-PAGE confirmed the expression of scFv-3, scFv-16, and scFv-24, although all three proteins accumulated exclusively as inclusion bodies (Figure S4). Following in vitro refolding, the His-scFv proteins were purified via affinity chromatography. SDS-PAGE verified that the expected molecular weights of the purified proteins, confirming the successful production of the recombinant scFv-3, scFv-16, and scFv-24 (Figure 5). Protein concentrations were quantified using a BCA assay. The binding of the three scFv proteins to His-tagged LEL was assessed by ELISA (Figure 6A–C). Apparent affinity constants for the His-LEL antigen were estimated by competitive ELISA with two antigen concentrations, using the Friguet calculation method [35]. Among the characterized clones, scFv-16 showed the highest apparent affinity, with a Kaff of 4.20 × 10^8^ M^−1^ (corresponding to a KD of 2.38 nM). scFv-16 was therefore selected for all subsequent analyses (Figure S5).

### 3.6. Binding Analysis of scFv-16 to TSPAN-1

**Figure 7:**
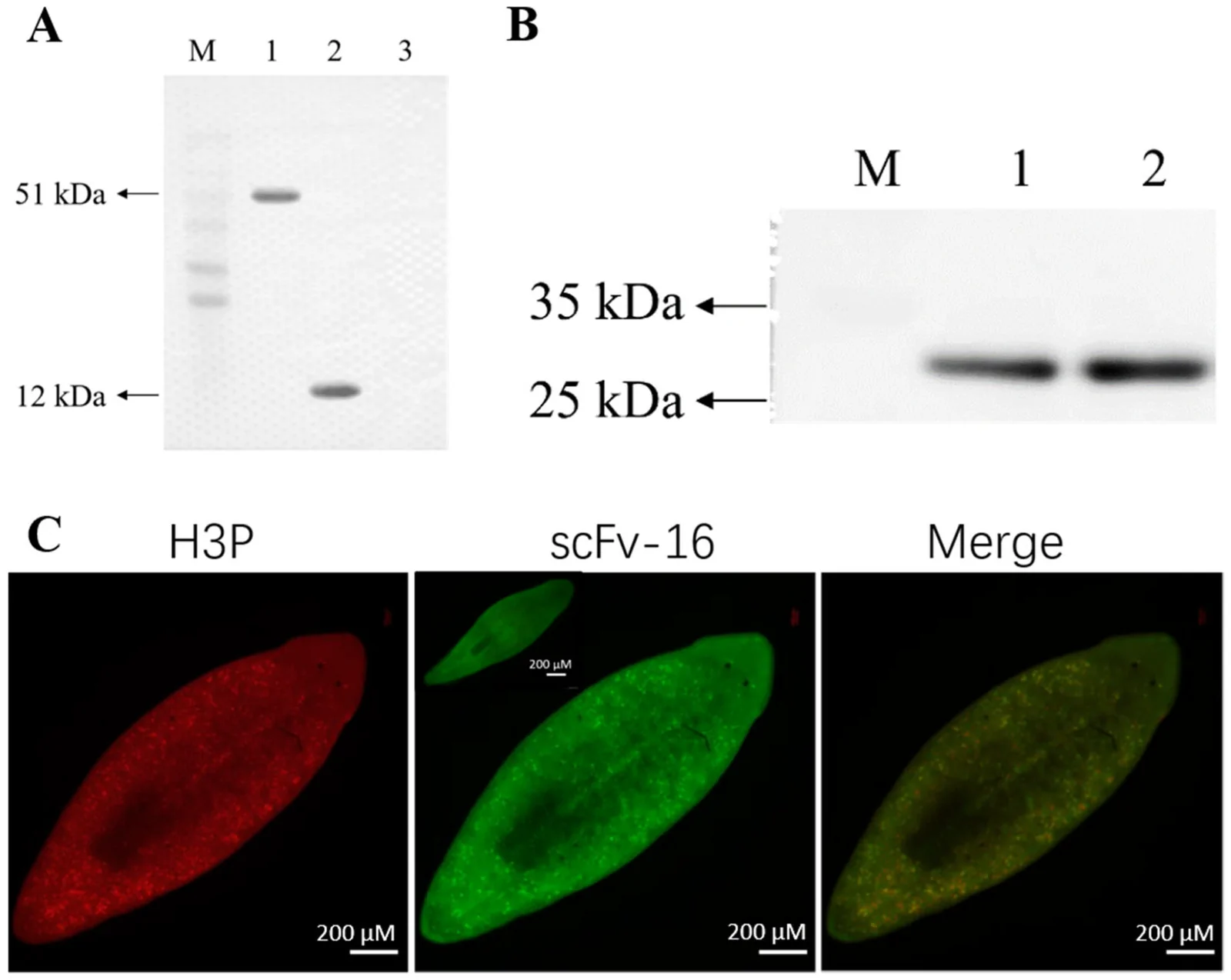
Specific binding analysis of scFv-16 to TSPAN-1. (**A**) Western blotting showing that scFv-16 recognized MBP-LEL (51 kDa) and His-LEL (12 kDa), but not the control MBP (46 kDa). (**B**) Western blotting of total protein from *Schmidtea mediterranea* with purified scFv-16 as the primary antibody; a single band appeared at approximately 28 kDa, consistent with the theoretical molecular weight of planarian TSPAN-1. (**C**) Immunofluorescence staining of whole-mount planarian tissue. Green signals (scFv-16, detected with an FITC-conjugated anti-His-tag antibody) overlapped with H3P-positive neoblasts (red). No specific fluorescence was observed when the primary antibody was omitted (inset, Green).

First, the specific binding of scFv-16 to purified recombinant antigens was evaluated by Western blotting. MBP-LEL (51 kDa), His-LEL (12 kDa), and a control MBP (46 kDa) were probed using scFv-16 as the primary antibody. scFv-16 specifically recognized bands at 51 kDa and 12 kDa, corresponding to MBP-LEL and His-LEL, respectively, whereas no signal was detected for the MBP control (Figure 7A). These results demonstrate that scFv-16 specifically recognizes the LEL epitopes TSPAN-1 without cross-reactivity to the MBP tag.

Whether scFv-16 also recognizes endogenous TSPAN-1 was examined. Total protein was extracted from *Schmidtea mediterranea* and probed by Western blotting with purified scFv-16 as the primary antibody (diluted 1:1000). A single band appeared at approximately 28 kDa, consistent with the theoretical molecular weight of planarian TSPAN-1 (Figure 7B).

To further validate the binding of scFv-16 to endogenous TSPAN-1 on planarian neoblasts, immunofluorescence staining was performed on planarian tissue. Whole-mount planarian tissue was fixed, methanol-dehydrated, and permeabilized with 1% SDS. The tissue was incubated with the purified scFv proteins and rabbit anti-H3P (phospho-histone H3 Ser10), a marker of dividing neoblasts, followed by detection with an FITC-conjugated anti-His-tag secondary antibody and an Alexa Fluor 568-conjugated anti-rabbit secondary antibody. The specimens were imaged with an inverted fluorescence microscope (Figure 7B). Collectively, these findings are consistent with specific binding of scFv-16 to endogenous TSPAN-1 in planarian neoblasts.

## 4. Discussion

Antibody-based fluorescence- and magnetic-activated cell sorting (FACS and MACS) have become widely used tools for isolating target subpopulations from heterogeneous cell populations [38], driving fundamental advances in stem cell biology [39], regenerative medicine [40], tumor microenvironment research [41], and developmental biology [42]. Planarian adult stem cells, known as neoblasts, underlie the organism’s exceptional whole-body regenerative capacity, making them an ideal model for studying tissue homeostasis and organ regeneration [14,43,44]. However, long-term in vitro expansion of neoblasts without loss of stemness remains a major challenge [10]. Overcoming this bottleneck necessitates the isolation of highly pure and viable starting cell populations [45]. Conventional enrichment of neoblasts from heterogeneous planarian cell suspensions has relied on DNA-binding fluorescent dyes such as Hoechst 33342. These approaches, however, frequently cause DNA damage and yield suboptimal sorting purity [46,47,48].

In contrast, antibody-based sorting targeting specific cell surface markers offers distinct advantages. This strategy markedly enhances the purity of the isolated subpopulation and enables non-destructive enrichment of live cells, thereby preserving their viability and proliferative potential [17,49]. It therefore provides a practical basis for elucidating microenvironmental signals and optimizing in vitro culture systems.

Recent studies have identified the transmembrane protein Tetraspanin-1 (TSPAN-1) as a specific surface marker of planarian neoblasts [17]. Transplantation of single TSPAN-1 positive cells into lethally irradiated planarians rescues the recipients by reconstituting all tissues and regenerating the entire organism. Phage display technology provides a simple and efficient platform for antibody engineering, owing to its operational simplicity and rapid screening capacity [25]. In this study, we employed this approach to construct a murine immune scFv phage display library directed against TSPAN-1 and screened for specific anti-TSPAN-1 scFvs. Our long-term goal is to develop a novel tool for the in vitro isolation of planarian stem cells.

Despite its solvent-exposed topology and enrichment in hydrophilic residues, the large extracellular loop (LEL) of TSPAN-1 tends to aggregate into inclusion bodies upon heterologous expression in *Escherichia coli*. To exploit the solubility-enhancing properties of the maltose-binding protein (MBP) tag, we generated two recombinant constructs: MBP-LEL for immunization and His-LEL for phage display bio-panning. MBP-LEL was selected as the immunogen because it affords high expression yields at low cost, whereas His-LEL served as the target antigen for screening a single-chain variable fragment (scFv) phage library across four rounds of bio-panning. This strategy yielded an approximately 100-fold increase in phage output across the four rounds, from which multiple candidate anti-TSPAN-1 clones were obtained. Affinity measurements by the Friguet method revealed an apparent affinity constant (Kaff) of 4.20 × 10^8^ M^−1^ for scFv-16 binding to His-LEL (corresponding to a KD of 2.38 nM), the highest among the characterized clones. This low-nanomolar affinity is encouraging for an immature scFv derived from an immune library, and the clone is expected to be applicable to standard antibody-based assays such as Western blotting and immunofluorescence. It should be noted that our immunofluorescence staining was performed on tissue that had been fixed, methanol-dehydrated, and permeabilized with 1% SDS; therefore, these data do not demonstrate binding of scFv-16 to the extracellular domain of native-conformation TSPAN-1 on intact cells, and further validation on live cells will be required. Its potential for live-cell applications, including FACS and MACS, will be evaluated in future studies [50].

Recombinant expression of scFv-16 in *E. coli* still results in inclusion body formation. Although soluble scFv can be recovered via in vitro refolding, this process may partially compromise antigen-binding activity, and its binding affinity could be further enhanced through systematic in vitro maturation. Therefore, future studies should explore eukaryotic expression platforms and integrate phage-based affinity maturation to further optimize both the affinity and biological functionality of the selected antibodies.

## 5. Conclusions

Long-term in vitro culture of neoblasts while maintaining their stemness remains a major challenge, and antibody-based sorting offers an effective strategy for the non-destructive enrichment of live cells. In this study, we constructed a murine immune scFv phage display library targeting TSPAN-1, a cell-surface marker of planarian neoblasts. Because the large extracellular loop of TSPAN-1 tends to form inclusion bodies upon heterologous expression in *Escherichia coli*, we generated an MBP-fused protein (MBP-LEL) as the immunogen and a His-tagged LEL protein (His-LEL) produced in a eukaryotic expression system as the antigen for bio-panning. Four rounds of panning yielded an approximately 100-fold increase in phage output and several candidate anti-TSPAN-1 clones; among the characterized clones, the lead clone scFv-16 bound His-LEL with an apparent affinity constant (Kaff) of 4.20 × 10^8^ M^−1^ (corresponding to a KD of 2.38 nM) as determined by the Friguet method. This nanomolar affinity is encouraging for an immature scFv derived from an immune library, and scFv-16 is expected to be applicable to standard antibody-based assays such as Western blotting and immunofluorescence. Its potential for live-cell applications, including FACS and MACS, will be evaluated in future studies.

## Figures and Tables

**Table 1:** Enrichment of specific binding phages for each bio-panning round.

Selection Round	Targets (μg/mL)	Wash Times (%Tween-20)	Input Phage (CFU/mL)	Output Phage (CFU/mL)
1	50	10 (0.1%)	1 × 10^9^	2.5 × 10^3^
2	25	15 (0.1%)	1 × 10^9^	8.6 × 10^3^
3	10	20 (0.5%)	1 × 10^9^	6.9 × 10^4^
4	5	25 (0.5%)	1 × 10^9^	2.7 × 10^5^

## Data Availability

The original contributions presented in this study are included in the article. Further inquiries can be directed to the corresponding authors.

## Supplementary Materials

The following supporting information can be downloaded at: https://www.mdpi.com/article/10.3390/biology15211928/s1, Supplementary Table S1: Information of primers used in this study; Figure S1: Agarose gel electrophoresis of *TSPAN-1* PCR products. Lane M, DL2000 DNA marker; lanes 1–2, *TSPAN-1* amplicons; Figure S2: Determination of the anti-TSPAN-1 phage-displayed scFv library capacity. (**A**,**B**) Plates from 10-2 and 10-3 dilutions, each yielding >500 colonies (too numerous to count). (**C**) Plate from the 10-4 dilution yielding 386 well-isolated colonies. The library titer was calculated as 3.86 × 10^7^ CFU/mL (plating volume, 100 μL); Figure S3: Identification of positive clones following bio-panning. (**A**) Colony PCR screening of 60 randomly picked transformants harboring pCANTAB5E-scFv phagemid. M, DL2000 DNA marker; lanes 1–60, PCR amplicons (900 bp). (**B**) Restriction analysis of candidate recombinant phagemids with *Sfi*I and *Not*I. M, DL2000 DNA marker; lanes 1–2, pCANTAB5E-scFv-3; lanes 3–4, pCANTAB5E-scFv-16; lanes 5–6, pCANTAB5E-scFv-24. Arrowhead indicates the excised scFv insert (750 bp); Figure S4: SDS-PAGE analysis of scFv solubility upon induced expression. (**A**) scFv-3(Lanes 1–2) and scFv-16 (lanes 3–4). M, protein marker; lanes 1 and 3, supernatants of sonicated *E. coli* lysates; lanes 2 and 4, pellets of sonicated *E. coli* lysates. (**B**) scFv-24. M, protein marker; lane 1, soluble fraction; lane 2, insoluble fraction. The scFv proteins were predominantly detected in the insoluble fractions, suggesting inclusion body formation; Figure S5: Sequence analysis of scFv-16 (VH-Linker-VL).

## References

[1] Zheng W., Peng B., Chen Z., Huang K., Qi Y., Niu H., Shen H., Wu J., Jiao J., Du P. (2026). Continuous modeling of primate embryogenesis from totipotency to early organogenesis. Cell.

[2] Qi L., He Y., Sviercovich A., Mei X., Chen E., Xia Y., Conboy M.J., Conboy I.M., Stahl A. (2026). Human microphysiological systems of aging recreate the in vivo process expediting evaluation of anti-geronic strategies. Nat. Biomed. Eng..

[3] Li J., Wang S., Yuan J., Mao X., Wang X., Zhang L., Dong Q., Chen Z., Wang Y., Tang N. (2025). Tissue regeneration: Unraveling strategies for resolving pathological fibrosis. Cell Stem Cell.

[4] Zhou M., Brinson D., Lei C., Karoubi G. (2025). Recent Advancements in the Generation and Application of Therapeutic Cell Populations for Lung Epithelial Repair. J. Tissue Eng. Regen. Med..

[5] Du P., Wu J. (2024). Hallmarks of totipotent and pluripotent stem cell states. Cell Stem Cell.

[6] Poss K.D., Tanaka E.M. (2024). Hallmarks of regeneration. Cell Stem Cell.

[7] Wagner D.E., Wang I.E., Reddien P.W. (2011). Clonogenic neoblasts are pluripotent adult stem cells that underlie planarian regeneration. Science.

[8] King H.O., Owusu-Boaitey K.E., Fincher C.T., Reddien P.W. (2024). A transcription factor atlas of stem cell fate in planarians. Cell Rep..

[9] Cheng X., Li Y., Guo Q., Tian Q., Zhang Y., Fang H., Zhang S., Guo Y. (2019). A planarian RPS3 homolog is critical to the modulation of planarian regeneration. Gene.

[10] Dai X., Li X., Tyshkovskiy A., Zuckerman C., Cheng N., Lin P., Paris D., Qureshi S., Kruglyak L., Mao X. (2025). Regeneration leads to global tissue rejuvenation in aging sexual planarians. Nat. Aging.

[11] Lei K., Zhang W., Chen J., McKinney S.A., Ross E.J., Lee H.C., Sánchez Alvarado A. (2023). Pluripotency retention and exogenous mRNA introduction in planarian stem cells in culture. iScience.

[12] Niu K., Xu H., Xiong Y.Z., Zhao Y., Gao C., Seidel C.W., Pan X., Ying Y., Lei K. (2021). Canonical and early lineage-specific stem cell types identified in planarian SirNeoblasts. Cell Regen..

[13] Molina M.D., Cebrià F. (2021). Decoding Stem Cells: An Overview on Planarian Stem Cell Heterogeneity and Lineage Progression. Biomolecules.

[14] Rink J.C. (2013). Stem cell systems and regeneration in planaria. Dev. Genes. Evol..

[15] Eisenhoffer G.T., Kang H., Sánchez Alvarado A. (2008). Molecular analysis of stem cells and their descendants during cell turnover and regeneration in the planarian Schmidtea mediterranea. Cell Stem Cell.

[16] Reddien P.W., Oviedo N.J., Jennings J.R., Jenkin J.C., Sánchez Alvarado A. (2005). SMEDWI-2 is a PIWI-like protein that regulates planarian stem cells. Science.

[17] Zeng A., Li H., Guo L., Gao X., McKinney S., Wang Y., Yu Z., Park J., Semerad C., Ross E. (2018). Prospectively Isolated Tetraspanin(+) Neoblasts Are Adult Pluripotent Stem Cells Underlying Planaria Regeneration. Cell.

[18] Seigneuret M., Delaguillaumie A., Lagaudrière-Gesbert C., Conjeaud H. (2001). Structure of the tetraspanin main extracellular domain. A partially conserved fold with a structurally variable domain insertion. J. Biol. Chem..

[19] Kovalenko O.V., Metcalf D.G., DeGrado W.F., Hemler M.E. (2005). Structural organization and interactions of transmembrane domains in tetraspanin proteins. BMC Struct. Biol..

[20] Umeda R., Satouh Y., Takemoto M., Nakada-Nakura Y., Liu K., Yokoyama T., Shirouzu M., Iwata S., Nomura N., Sato K. (2020). Structural insights into tetraspanin CD9 function. Nat. Commun..

[21] Zhou Z., Yang Z., Zhou L., Yang M., He S. (2023). The versatile roles of testrapanins in cancer from intracellular signaling to cell-cell communication: Cell membrane proteins without ligands. Cell Biosci..

[22] Jahandar-Lashaki S., Farajnia S., Faraji-Barhagh A., Hosseini Z., Bakhtiyari N., Rahbarnia L. (2025). Phage Display as a Medium for Target Therapy Based Drug Discovery, Review and Update. Mol. Biotechnol..

[23] Hoogenboom H.R. (2002). Overview of antibody phage-display technology and its applications. Methods Mol. Biol..

[24] Finlay W.J., Bloom L., Grant J., Franklin E., Shúilleabháin D.N., Cunningham O. (2017). Phage Display: A Powerful Technology for the Generation of High-Specificity Affinity Reagents from Alternative Immune Sources. Methods Mol. Biol..

[25] Hutchings C.J., Sato A.K. (2024). Phage display technology and its impact in the discovery of novel protein-based drugs. Expert. Opin. Drug Discov..

[26] Unkauf T., Miethe S., Fühner V., Schirrmann T., Frenzel A., Hust M. (2016). Generation of Recombinant Antibodies Against Toxins and Viruses by Phage Display for Diagnostics and Therapy. Adv. Exp. Med. Biol..

[27] Chan D.T.Y., Groves M.A.T. (2021). Affinity maturation: Highlights in the application of in vitro strategies for the directed evolution of antibodies. Emerg. Top. Life Sci..

[28] Frenzel A., Schirrmann T., Hust M. (2016). Phage display-derived human antibodies in clinical development and therapy. MAbs.

[29] Steinwand M., Droste P., Frenzel A., Hust M., Dübel S., Schirrmann T. (2014). The influence of antibody fragment format on phage display based affinity maturation of IgG. MAbs.

[30] Schirrmann T., Meyer T., Schütte M., Frenzel A., Hust M. (2011). Phage display for the generation of antibodies for proteome research, diagnostics and therapy. Molecules.

[31] Guo Y., Yu M., Jing N., Zhang S. (2018). Production of soluble bioactive mouse leukemia inhibitory factor from Escherichia coli using MBP tag. Protein Expr. Purif..

[32] Fields C., O’Connell D., Xiao S., Lee G.U., Billiald P., Muzard J. (2013). Creation of recombinant antigen-binding molecules derived from hybridomas secreting specific antibodies. Nat. Protoc..

[33] McGuire M.J., Li S., Brown K.C. (2009). Biopanning of phage displayed peptide libraries for the isolation of cell-specific ligands. Methods Mol. Biol..

[34] Saroha P., Patil R.S., Rathore A.S. (2025). Recent advancements in soluble expression of recombinant antibody fragments in microbial host systems. Prep. Biochem. Biotechnol..

[35] Friguet B., Chaffotte A.F., Djavadi-Ohaniance L., Goldberg M.E. (1985). Measurements of the true affinity constant in solution of antigen-antibody complexes by enzyme-linked immunosorbent assay. J. Immunol. Methods.

[36] Newmark P.A., Sánchez Alvarado A. (2000). Bromodeoxyuridine specifically labels the regenerative stem cells of planarians. Dev. Biol..

[37] Wenemoser D., Reddien P.W. (2010). Planarian regeneration involves distinct stem cell responses to wounds and tissue absence. Dev. Biol..

[38] Sutermaster B.A., Darling E.M. (2019). Considerations for high-yield, high-throughput cell enrichment: Fluorescence versus magnetic sorting. Sci. Rep..

[39] Tripathi H., Peng H., Donahue R., Chelvarajan L., Gottipati A., Levitan B., Al-Darraji A., Gao E., Abdel-Latif A., Berron B.J. (2020). Isolation Methods for Human CD34 Subsets Using Fluorescent and Magnetic Activated Cell Sorting: An In Vivo Comparative Study. Stem Cell Rev. Rep..

[40] Guo J., Cairns B.R. (2019). Isolation and Enrichment of Spermatogonial Stem Cells From Human Testis Tissues. Curr. Protoc. Stem Cell Biol..

[41] Müller S., Kohanbash G., Liu S.J., Alvarado B., Carrera D., Bhaduri A., Watchmaker P.B., Yagnik G., Di Lullo E., Malatesta M. (2017). Single-cell profiling of human gliomas reveals macrophage ontogeny as a basis for regional differences in macrophage activation in the tumor microenvironment. Genome Biol..

[42] Takata K., Kozaki T., Lee C.Z.W., Thion M.S., Otsuka M., Lim S., Utami K.H., Fidan K., Park D.S., Malleret B. (2020). Induced-Pluripotent-Stem-Cell-Derived Primitive Macrophages Provide a Platform for Modeling Tissue-Resident Macrophage Differentiation and Function. Immunity.

[43] Ivankovic M., Haneckova R., Thommen A., Grohme M.A., Vila-Farré M., Werner S., Rink J.C. (2019). Model systems for regeneration: Planarians. Development.

[44] Elliott S.A., Sánchez Alvarado A. (2013). The history and enduring contributions of planarians to the study of animal regeneration. Wiley Interdiscip. Rev. Dev. Biol..

[45] Hall R.N., Weill U., Drees L., Leal-Ortiz S., Li H., Khariton M., Chai C., Xue Y., Rosental B., Quake S.R. (2022). Heterologous reporter expression in the planarian Schmidtea mediterranea through somatic mRNA transfection. Cell Rep. Methods.

[46] Hayashi T., Asami M., Higuchi S., Shibata N., Agata K. (2006). Isolation of planarian X-ray-sensitive stem cells by fluorescence-activated cell sorting. Dev. Growth Differ..

[47] Peiris T.H., García-Ojeda M.E., Oviedo N.J. (2016). Alternative flow cytometry strategies to analyze stem cells and cell death in planarians. Regeneration.

[48] Ishida M., Kuroki Y., Agata K. (2023). Establishment of a new method to isolate viable x-ray-sensitive cells from planarian by fluorescence-activated cell sorting. Dev. Growth Differ..

[49] Manohar S.M., Shah P., Nair A. (2021). Flow cytometry: Principles, applications and recent advances. Bioanalysis.

[50] Ahmad Z.A., Yeap S.K., Ali A.M., Ho W.Y., Alitheen N.B., Hamid M. (2012). scFv antibody: Principles and clinical application. Clin. Dev. Immunol..

