# Supplementary material for: Isolation and Characterization of a High-Affinity Anti-TSPAN-1 scFv Antibody from a Phage-Display Library for Planarian Neoblast Targeting
Source: Biology (Basel). 2026 Oct 24;15(21):1928. doi: 10.3390/biology15211928 (PMC13761934; doi:10.3390/biology15211928)
Supplement: Supplementary file 1 [file biology-15-01928-s001.zip › Supplementary Figure.pdf]

Supplementary Figure

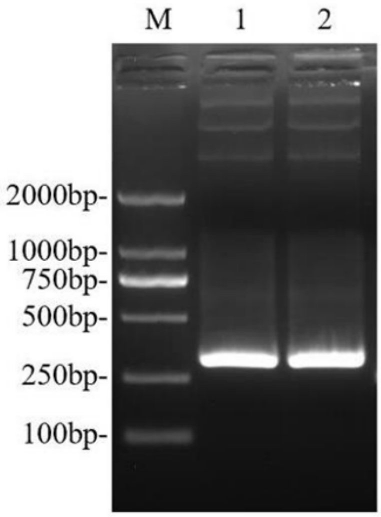

Figure S1. Agarose gel electrophoresis of *TSPAN-1* PCR products. Lane M, DL2000 DNA marker; lanes 1–2, *TSPAN-1* amplicons.

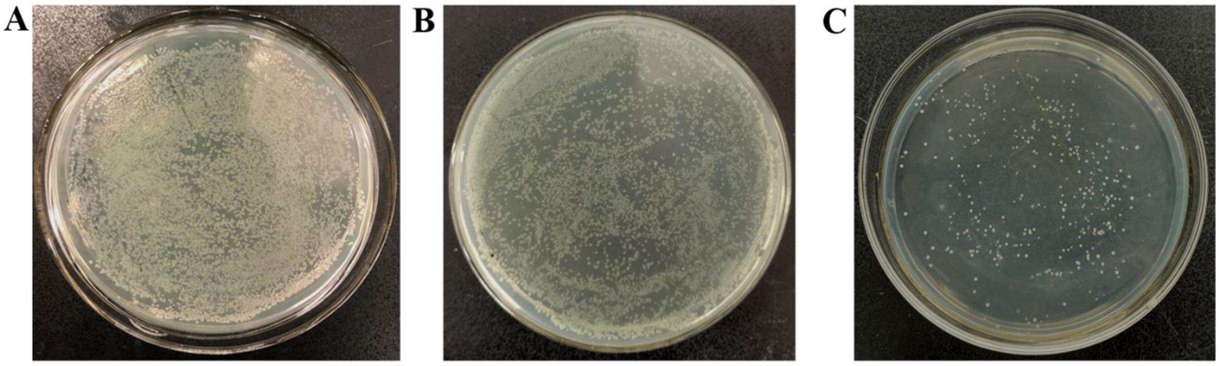

Figure S2. Determination of the anti-TSPAN-1 phage-displayed scFv library capacity. (A–B) Plates from 10^{-2} and 10^{-3} dilutions, each yielding >500 colonies (too numerous to count). **(C)** Plate from the 10^{-4} dilution yielding 386 well-isolated colonies. The library titer was calculated as 3.86×10^7 CFU/mL (plating volume, 100 μL).

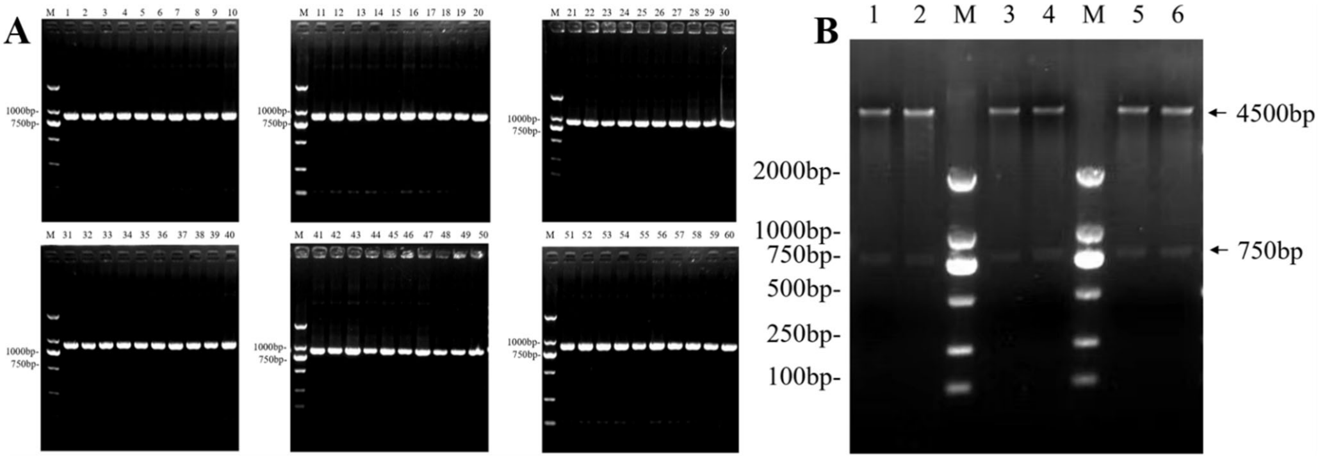

Figure S3. Identification of positive clones following bio-panning. (A) Colony PCR screening of 60 randomly picked transformants harboring pCANTAB5E-scFv phagemid. M, DL2000 DNA marker; lanes 1–60, PCR amplicons (900 bp). **(B)** Restriction analysis of candidate recombinant phagemids with *Sfi*I and *Not*I. M, DL2000 DNA marker; lanes 1–2, pCANTAB5E-scFv-3; lanes 3–4, pCANTAB5E-scFv-16; lanes 5–6, pCANTAB5E-scFv-24. Arrowhead indicates the excised scFv insert (750 bp).

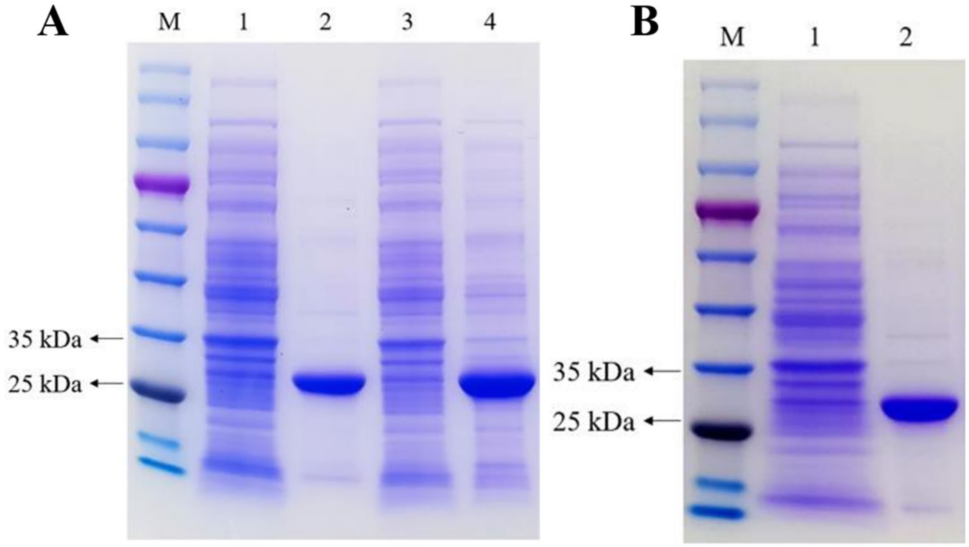

Figure S4. SDS-PAGE analysis of scFv solubility upon induced expression. (A) scFv-3(Lanes 1–2) and scFv-16 (lanes 3–4). M, protein marker; lanes 1 and 3, supernatants of sonicated *E. coli* lysates; lanes 2 and 4, pellets of sonicated *E. coli* lysates. **(B)** scFv-24. M, protein marker; lane 1, soluble fraction; lane 2, insoluble fraction. The scFv proteins were predominantly detected in the insoluble fractions, suggesting inclusion body formation.

Figure S5. Sequence analysis of scFv-16 (VH-Linker-VL)

QVQLQQSGPQLIRPGASVKISCKATGYTFSTYWIEWVKQRPGHGLEWIGEILPGSGSTNYN
EKFKGKATFTADTSSNTAYMQLSSLTSEDSAVYYCARRRWGNYVGYFDYWGQGTTVTVS
SGGGGSGGGGSGGGGSDIELTQSPAIMSASPGEKVTMTCRASSTVSSSYLHWYQQKSGAS
PKLWIYSTSNLASGVPARFSGSGSGTSYSLTISQMEAEDAATYHCQQWSSYPTFGTGTKLEI
KR
